# Epidemiological status and associated factors of frailty and pre-frailty in older adults with asthma in China: A national cross-sectional study

**DOI:** 10.3389/fpubh.2023.1136135

**Published:** 2023-03-03

**Authors:** Xue-zhai Zeng, Ling-bing Meng, Na Jia, Jing Shi, Chi Zhang, Ying-ying Li, Xing Hu, Jia-bin Hu, Jian-yi Li, Di-shan Wu, Hui Li, Xin Qi, Hua Wang, Qiu-xia Zhang, Juan Li, De-ping Liu

**Affiliations:** ^1^Department of Cardiology, Beijing Hospital, National Center of Gerontology, Institute of Geriatric Medicine, Chinese Academy of Medical Sciences, Beijing, China; ^2^Department of Geriatrics, Beijing Hospital, National Center of Gerontology, Institute of Geriatric Medicine, Chinese Academy of Medical Sciences, Beijing, China; ^3^The Key Laboratory of Geriatrics, Beijing Institute of Geriatrics, Institute of Geriatric Medicine, Chinese Academy of Medical Sciences, Beijing Hospital/National Center of Gerontology of National Health Commission, Beijing, China; ^4^Health Service Department of the Guard Bureau of the Joint Staff Department, Beijing, China; ^5^China Research Center on Ageing, Beijing, China; ^6^Institute of Psychology, Chinese Academy of Sciences, Beijing, China

**Keywords:** asthma, China, frailty, older adults, pre-frailty, prevalence

## Abstract

**Objective:**

There are few studies on the prevalence and factors associated with frailty and pre-frailty in older adults with asthma worldwide. The aim of this study was to examine the epidemiological status and factors associated with frailty and pre-frailty in older adults with asthma in China.

**Research design and methods:**

Data were obtained from the Sample Survey of Aged Population in Urban and Rural China in 2015, a nationwide cross-sectional survey covering 224,142 older people aged 60 years or older in 31 provinces/autonomous regions/municipalities in mainland China. We performed frailty and pre-frailty assessments using the frailty index, and the diagnosis of asthma in the older adults was self-reported based on the history of the physician's diagnosis.

**Results:**

Nine thousand four hundred sixteen older adults with asthma were included in the study. The age-sex standardized prevalence of frailty and pre-frailty in Chinese older adults with asthma was 35.8% (95% CI 34.8%−36.7%) and 54.5% (95% CI 53.5%−55.5%). Multinomial logistic regression analysis showed that increased age, female, illiteracy, living alone, poor economic status, ADL disability, comorbid chronic diseases, previous hospitalization in the past year, and residence in northern China were associated with frailty and pre-frailty in older adults with asthma.

**Conclusion:**

The prevalence of frailty and pre-frailty in Chinese older adults with asthma is very high, and assessment of frailty should become routine in the management of older adults with asthma. Appropriate public health prevention strategies based on identified risk factors for frailty in older adults with asthma should be developed to reduce the burden of frailty in Chinese older adults with asthma.

## Introduction

Asthma is a common non-communicable lung disease, with an aging population, the number of older people suffering from asthma is increasing. Epidemiological data show that the prevalence of asthma in people aged 65 years or over is 4%−15% (1–3). The 2017 global burden of disease study revealed asthma to be the second most prevalent chronic respiratory disease after chronic obstructive respiratory disease and the second leading cause of death from chronic respiratory disease (4). Asthma poses a serious threat to the quality of life and health of older people, and increases the use and burden of healthcare resources on society (5). Frailty is a clinical syndrome characterized by reduced physiological reserve and multisystem dysregulation, which limits the body's ability to respond to internal and external stresses and maintain the stability of the internal environment, increasing the body's susceptibility to stressful events (6–8). The weighted prevalence of frailty in older people in the community is 11% (range 4%−59%) (9). Frail older people are at significantly increased risk of falls, delirium, incapacity, hospitalization and death, and increase the burden on society's healthcare resources (8, 10, 11). There are few large-scale studies in the world on frailty in older adults with asthma. The paucity of research on frailty in older patients with asthma has led to many challenges in the comprehensive management of asthma in older adults. On top of the existing medical care for older adults with asthma, frailty assessment can provide additional valuable information and help clinicians choose better medical care for their patients. This study applied data from the 2015 Sample Survey of Aged Population in Urban and Rural China (SSAPUR) to analyse the prevalence of frailty and pre-frailty and their associated factors in older adults with asthma in China. The aim was to provide information to reduce the decline in cognitive and physical functioning and to prevent frailty and disability in older people with asthma.

## Methods

### Study design and participants

**Figure 1:**
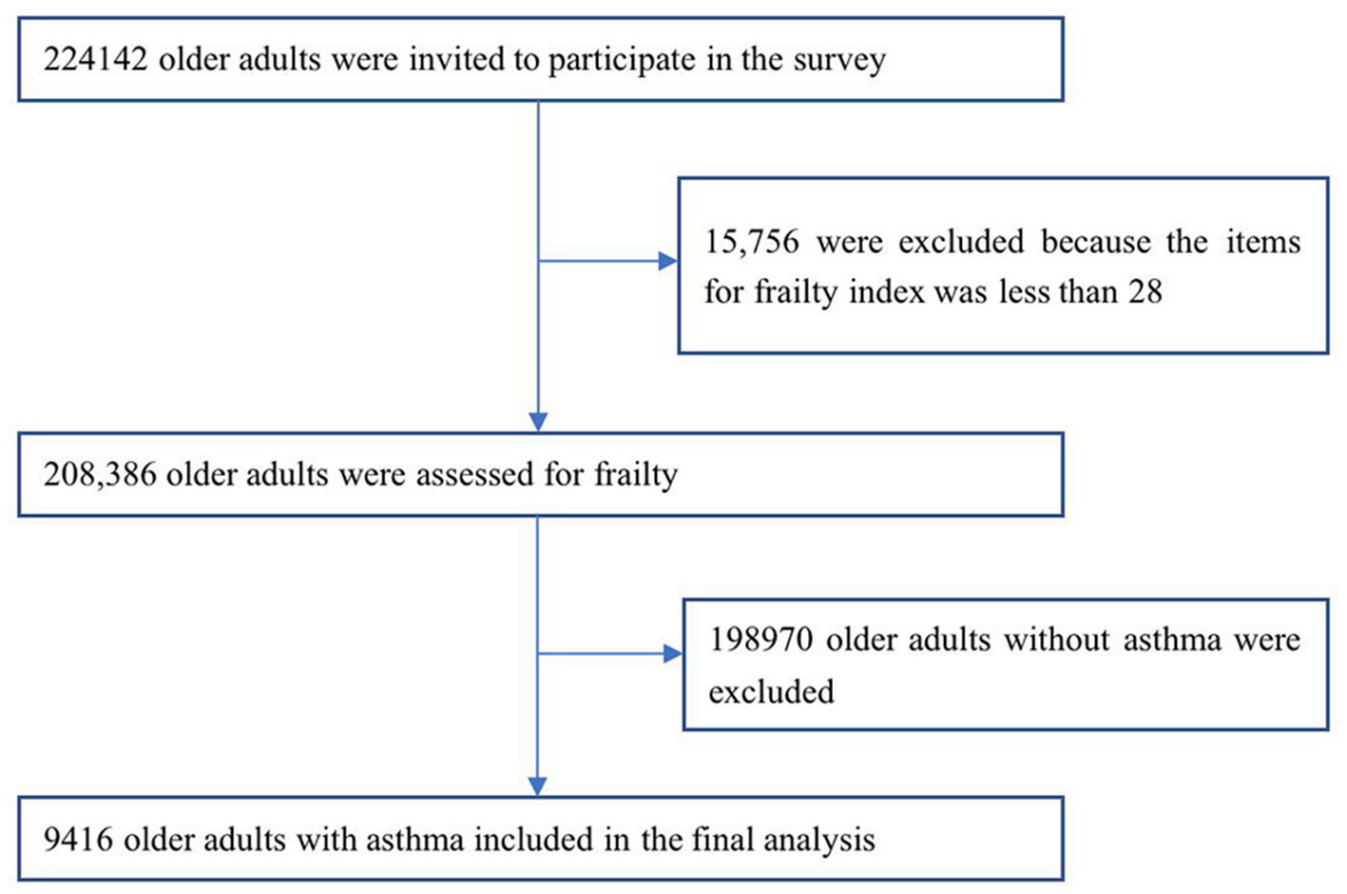
Flowchart of study participants on frailty and pre-frailty prevalence in older adults with asthma in China.

Data were obtained from the 4th SSAPUR, a cross-sectional study of 224,142 older adults aged 60 years or older in 31 provinces, autonomous regions, and municipalities in mainland China in 2015. The survey used a stratified, multi-stage, proportional probability sampling by size and equal probability sampling design in the final stage. The sampling proportion was ~1 in 1,000 of the national older population in 2015, and the sample obtained was self-weighted to ensure national representativeness. The sample number was assigned based on the proportion of the older population in each province, autonomous regions, and municipalities of the country, after that the number of counties, towns and communities sampled was determined and 462 counties were selected. Based on PPS sampling, four towns were selected from each county and four communities (villages or neighborhood councils) were selected from each town. Finally, 30 older adults were selected from each community using equidistant sampling. Data on the living conditions of older adults were collected through household interviews and questionnaires. More information on the design and sampling methods of the 4th SSAPUR study has been reported in previous studies (12–14). We used the frailty index (FI) for frailty and pre-frailty assessment, and 15,756 (7.0%) older adults were excluded because the number of constructed FI items was <28. Of the 208,386 older adults, 9,416 older adults with asthma, determined on the basis of a self-reported history of diagnosis by a physician, were included in our study (Figure 1). The study protocol was approved by the National Bureau of Statistics (No. [2014] 87) and the Ethics Committee of Beijing Hospital (2021BJYYEC-294-01). Written informed consent was provided by all participants.

### Demographics

Demographic characteristics: age, sex, education, marital status, ethnicity, residence, living status, health checkup within the past year, hospitalization within the past year, financial status, ease of medical reimbursement, activities of daily living (ADL) disability (Inability to do one or more of the following: bathing, dressing, toileting, getting in and out of bed, eating and moving around the room is considered a disability), comorbid chronic diseases, and residence in southern or northern China.

### Identification and assignment of health deficit variables for FI

FI refers to the proportion of deficits that are present when a person undergoes a health assessment. We constructed FI following Searle's standard procedure (15). The FI items (*n* = 33, each subject needs at least 28/33 variables) were selected from the baseline questionnaires of demographic characteristics, physical health, physical functioning, lifestyle, social activity, and mental health status. The variables included eight items of basic activities of daily living (bathing, dressing, toileting, getting in and out of bed, eating, walking around the room, urinary incontinence, and fecal incontinence); 10 items focusing on chronic diseases included glaucoma/cataract, cardiovascular disease, hypertension, diabetes, gastric disease, bone and joint disease, chronic lung disease, asthma, malignancy, and reproductive system disease; two items focused on feelings of loneliness and happiness; three items focused on geriatric syndrome, including visual impairment, hearing impairment, and history of falls; five items focused on assistive devices (hearing aids, dentures, crutches, wheel-chairs, and adult diapers/nursing pads); three items focused on mobility (needing care from others in daily life, self-rated health status, and exercise); two items focused on social activity (regular leisure activities and regular public service activities). The FI was calculated by summing the number of deficits recorded for a patient and dividing this by the total number of possible deficits. The exact construction method has been described in our previous study (12). FI scores ≥0.25 are considered frailty, <0.12 are considered robust, and FI 0.12–0.25 are considered pre-frailty.

### Statistical analysis

We used SPSS 24.0 software for statistical analysis. Missing data were interpolated using the Markov chain Monte Carlo (MCMC) multiple fill method (15). The age-standardized prevalence of frailty and pre-frailty among Chinese older adults with asthma was calculated based on the weights established in our study. For continuous variables, we assessed the significance of differences by ANOVA or Student's t-test, and for categorical variables, we passed the χ^2^ test. Trends in prevalence of covariates were examined by using the Cochran-Armitage test. Multinomial regression analysis was used to identify factors associated with frailty and pre-frailty, including age group, gender, ethnicity, urban/rural, education level, marital status, living alone, economic status, health insurance, ease of medical reimbursement, comorbid chronic diseases, ADL disability, and residence in southern or northern China, with *p* < 0.05 being statistically significant.

## Results

From August 1, 2015 to August 31, 2015, 224,142 older adults aged 60 years or older were invited to participate in the fourth SSAPUR, of which 15,756 participants were excluded because fewer than 28 items were used to construct the FI. Nine thousand four hundred sixteen older adults with self-reported asthma out of 208,386, and the self-reported prevalence of asthma among older adults was 4.5%, with 4.8% prevalence in men and 4.3% prevalence in women. Demographics and frailty risk factors by frailty stage in older adults with asthma are shown in Table 1, demographics and frailty risk factors for older asthmatics by North and South are shown in Supplemental Table S1.

**Table 1:** Demographics of the Chinese adults aged 60 years or older with asthma in 2015, and related factors for frailty, by frailty stage.

	**Total (*n* = 9,416)**	**Men (*n* = 4,774)**	**Women (*n* = 4,642)**	***p*-value**	**Men**				**Women**			
					**Robust (546)**	**Pre-frailty (2,762)**	**Frailty (1,466)**	***p*** **for difference**	**Robust (383)**	**Pre-frailty (2,380)**	**Frailty (1,879)**	***p*** **for difference**
Proportional of participants	100%	50.7%	49.3%		11.4%	57.9%	30.7%	<0.001	8.3%	51.3%	40.5%	<0.001
Age (years)	71.9 ± 8.1	71.4 ± 7.9	72.3 ± 8.3	<0.001	69.8 ± 7.6	71.0 ± 7.6	72.8 ± 8.1	<0.001	70.6 ± 8.1	71.5 ± 7.9	73.7 ± 8.6	<0.001
**Age group**												
60–69	44.4%	45.5%	43.2%	<0.001	56.2%	47.4%	37.8%	<0.001	51.9%	47.2%	36.5%	<0.001
70–79	35.8%	36.9%	34.7%		30.8%	36.9%	39.4%		31.1%	34.3%	35.9%	
≥80	19.8%	17.6%	22.1%		13.0%	15.7%	22.8%		17.0%	18.5%	27.6%	
Urban or rural area (Urban)	41.4%	41.0%	41.9%	0.361	44.9%	40.2%	40.9%	0.130	47.3%	41.1%	41.7%	0.078
Education (illiteracy)	36.9%	19.0%	55.3%	<0.001	15.0%	18.8%	20.9%	0.010	45.7%	52.4%	60.9%	<0.001
**Marital status**												
Married	65.9%	76.7%	54.8%	<0.001	83.5%	76.6%	74.1%	<0.001	68.4%	58.8%	47.0%	<0.001
Widowed	31.4%	18.6%	44.7%		13.6%	17.8%	22.0%		31.6%	40.6%	52.4%	
Divorced	0.8%	1.1%	0.4%		1.1%	1.4%	0.8%		0	0.5%	0.5%	
Unmarried	1.9%	3.6%	0.1%		1.8%	4.2%	3.1%		0	0.1%	0.1%	
Ethnicity (non-Han)	7.0%	6.5%	7.6%	0.035	6.8%	6.4%	6.3%	0.940	9.1%	8.4%	6.1%	0.008
Living alone	16.2%	13.8%	18.5%	<0.001	5.0%	15.9%	24.6%	<0.001	6.2%	14.0%	16.4%	<0.001
Health checkup within 1 year	57.8%	58.2%	57.4%	0.469	56.8%	58.4%	58.3%	0.782	53.0%	59.9%	55.2%	0.002
Hospitalized within 1 year	44.6%	44.7%	44.4%	0.800	70.7%	58.3%	54.1%	<0.001	76.2%	59.2%	46.8%	<0.001
**Economic status**												
Very rich	0.8%	0.8%	0.7%	0.545	1.5%	0.8%	0.7%	<0.001	1.3%	0.7%	0.7%	<0.001
Rich	12.0%	12.1%	12.0%		16.9%	12.5%	9.7%		21.7%	12.8%	9.1%	
Adequate	55.1%	55.8%	54.4%		63.7%	57.7%	49.2%		57.4%	58.1%	49.0%	
Poor	26.6%	25.9%	27.2%		15.9%	25.1%	31.2%		18.3%	25.0%	31.9%	
Very poor	5.5%	5.4%	5.7%		2.0%	3.9%	9.2%		1.3%	3.4%	9.3%	
Medicare (no)	1.0%	1.0%	1.0%	0.810	0.9%	0.9%	1.0%	0.960	0.5%	0.9%	1.3%	0.268
**Convenience of medical cost reimbursement**												
Highly convenient	30.6%	30.2%	31.1%	0.555	36.6%	29.3%	29.5%	<0.001	28.7%	31.8%	30.5%	0.003
Convenient	43.4%	43.1%	43.6%		42.5%	44.0%	41.7%		50.9%	42.7%	43.3%	
Less convenient	18.9%	19.6%	18.2%		16.1%	20.2%	19.7%		15.9%	19.1%	17.7%	
Inconvenient	5.0%	5.0%	4.9%		3.1%	4.8%	6.2%		3.7%	4.1%	6.2%	
Highly inconvenient	2.1%	2.1%	2.2%		1.7%	1.7%	2.9%		0.8%	2.3%	2.3%	
Comorbidities (≥1)	92.0%	90.9%	93.2%	<0.001	48.5%	94.7%	99.6%	<0.001	53.3%	94.6%	99.5%	<0.001
ADL disability	7.8%	6.6%	9.1%	<0.001	0.2%	2.0%	17.8%	<0.001	0	2.0%	20.0%	<0.001
Living southern or northern China (Northern China)	25.3%	22.9%	27.7%	<0.001	15.4%	21.1%	29.1%	<0.001	21.9%	23.4%	34.4%	<0.001

ADL, activities of daily living.

The FI of older adults with asthma was gamma distributed with a statistical value of 0.075, *p* < 0.0001. The FI of older adults with asthma was 0.21 (0.11; ranging from 0.04–0.70), with 0.23 (0.12) for female asthmatics, which was higher than that of male asthmatics 0.20 (0.11; *z* = −11.686, *p* < 0.001).

The prevalence of frailty and pre-frailty in older adults with asthma was 35.5% and 54.6%, respectively, significantly higher than the prevalence of frailty (8.2%) and pre-frailty (46.4%) in older adults without asthma, both *p* < 0.0001. The prevalence of frailty was higher in female older adults with asthma (40.5%) than in males (30.7%), while the prevalence of pre-frailty was lower in female older adults with asthma (51.3%) than in males (57.9%), both *p* < 0.001. The age-sex standardized prevalence of frailty and pre-frailty among Chinese older adults with asthma was 35.8% (95% CI 34.8%−36.7%) and 54.5% (95% CI 53.5%−55.5%). The age-sex standardized prevalence of frailty and pre-frailty was 40.9% (95% CI 39.5%−42.3%) and 51.4% (95% CI 49.9%−52.8%) for women, and 30.8% (95% CI 29.5%−32.1%) and 57.5% (95% CI 56.1%−58.9%) for men.

**Figure 2:**
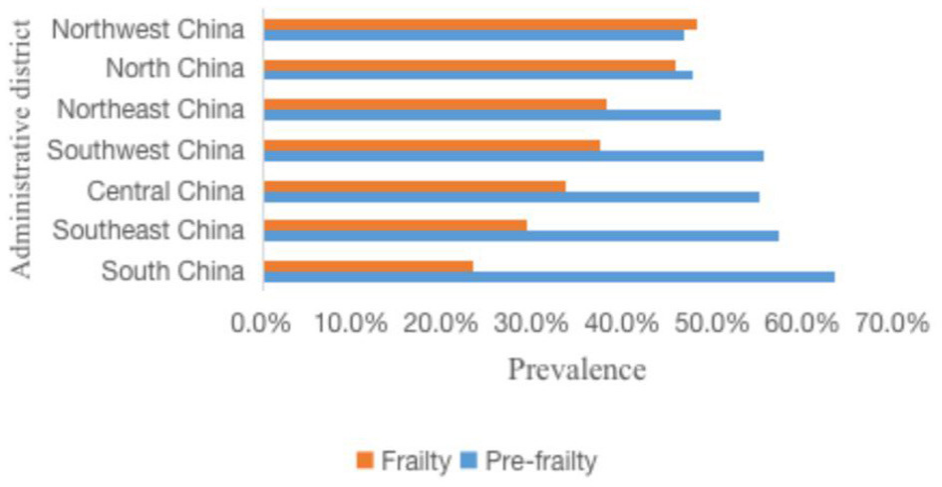
Prevalence of frailty and pre-frailty in older adults with asthma in different administrative regions.

The prevalence of frailty in older adults with asthma increased with age, from 29.7% in the 60–69 years age group to 45.8% in the ≥80 years age group. There was no difference in the prevalence of frailty between rural and urban older adults with asthma. The prevalence of frailty was higher in Han Chinese older adults with asthma than in non-Han Chinese, mainly in Han Chinese women than in non-Han Chinese women. Frailty was mostly seen in older adults with asthma who were illiterate, widowed, living alone, had been hospitalized in the past 1 year, had financial difficulties, had difficulties in reimbursing medical expenses, and had comorbid chronic diseases and disabilities. The prevalence of frailty was higher among older adults with asthma in northern China than in southern China (Table 2). The prevalence of frailty among older adults with asthma in the seven administrative regions of mainland China was highest in northwest China, followed by north China, then northeast China, and then southwest, central, southeast, and south China (Figure 2).

**Table 2:** Prevalence of frailty and pre-frailty among older adults with asthma in China in 2015.

	**All (*****n*** = **9,416)**			**Men (*****n*** = **4,774)**			**Women (*****n*** = **4,642)**		
	**Prevalence of pre-frail**	**Prevalence of frail**	***p*** **-value**	**Prevalence of pre-frail**	**Prevalence of frail**	***p*** **-value**	**Prevalence of pre-frail**	**Prevalence of frail**	***p*** **-value**
Proportional of participants	54.6%	35.5%	<0.001	57.9%	30.7%	<0.001	51.3%	40.5%	<0.001
**Age group**									
60–69	58.2%^a^	29.7%^a^	<0.001	60.3%^a^	25.5%^a^	<0.001	55.9%^a^	34.2%^a^	<0.001
70–79	54.4%^b^	37.1%^b^		57.7%^a^	32.8%^b^		50.7%^b^	41.9%^b^	
≥80	46.9%^c^	45.8%^c^		51.7%^b^	39.8%^c^		43.0%^c^	50.7%^c^	
**Urban or rural area**									
Urban	53.6%^a^	35.5%^a^	0.012	56.8%^a^	30.6%^a^	0.130	50.4%^a^	40.3%^a^	0.078
Rural	55.3%^a^	35.6%^a^		58.6%^a^	30.8%^a^		51.9%^a^	40.6%^a^	
**Education**									
Illiteracy	50.8%^a^	41.8%^a^	<0.001	57.2%^a^	33.8%^a^	0.010	48.6%^a^	44.6%^a^	<0.001
Non-illiteracy	56.8%^b^	31.9%^b^		58.0%^a^	30.0%^b^		54.6%^b^	35.4%^b^	
**Marital status**									
Married	56.7%^a^	31.7%^a^	<0.001	57.9%^a, b^	29.7%^a^	<0.001	55.0%^a^	34.7%^a^	<0.001
Widowed	49.3%^b^	44.2%^b^		55.3%^b^	36.3%^b^		46.6%^b^	47.5%^b^	
Divorced	64.9%^a, c^	27.0%^a^		68.5%^a, b^	20.4%^a, b^		55.0%^a, b^	45.0%^a, b^	
Unmarried	67.4%^c^	26.9%^a^		67.4%^a^	26.7%^a, b^		66.7%^a, b^	33.3%^a, b^	
**Ethnicity**									
Han	54.4%^a^	35.8%^a^	0.081	57.9%^a^	30.7%^a^	0.940	50.8%^a^	41.1%^a^	0.008
Non-Han	57.5%^a^	31.6%^b^		57.8%^a^	30.2%^a^		57.3%^b^	32.8%^b^	
**Living status**									
Living alone	50.4%^a^	46.2%^a^	<0.001	58.5%^a^	36.3%^a^	<0.001	44.1%^a^	53.7%^a^	<0.001
Not living alone	55.4%^b^	33.5%^b^		57.7%^a^	29.8%^b^		52.9%^b^	37.5%^b^	
**Health checkup within 1 year**									
No	53.0%^a^	36.6%^a^	0.017	57.6%^a^	30.6%^a^	0.782	48.3%^a^	42.6%^a^	0.002
Yes	55.8%^b^	34.8%^a^		58.0%^a^	30.8%^a^		53.5%^b^	38.9%^b^	
**Hospitalized within 1 year**									
No	57.8%^a^	29.2%^a^	<0.001	60.9%^a^	24.5%^a^	<0.001	54.6%^a^	34.1%^a^	<0.001
Yes	50.6%^b^	43.4%^b^		54.1%^b^	38.4%^b^		47.1%^b^	48.5%^b^	
**Economic status**									
Very rich	52.7%^a, b, c^	29.7%^a, b^	<0.001	55.0%^a, b^	25.0%^a, b^	<0.001	50.0%^a, b, c^	35.3%^a, b^	<0.001
Rich	57.0%^c^	27.6%^b^		59.5%^b^	24.6%^b^		54.5%^c^	30.6%^b^	
Adequate	57.4%^c^	31.7%^b^		59.8%^b^	27.1%^b^		54.8%^c^	36.5%^b^	
Poor	51.5%^b^	42.2%^a^		56.1%^b^	36.9%^a^		47.0%^b^	47.5%^a^	
Very poor	36.9%^a^	60.0%^c^		42.7%^a^	52.9%^c^		31.3%^a^	66.8%^c^	
**Medicare**									
Yes	54.6%^a^	35.5%^a^	0.388	57.9%^a^	30.7%^a^	0.960	51.3%^a^	40.4%^a^	0.268
No	50.5%^a^	41.9%^a^		56.5%^a^	32.6%^a^		44.7%^a^	51.1%^a^	
**Convenience of medical cost reimbursement**									
Highly convenient	54.3%^a, b^	34.9%^a^	<0.001	56.1%^a^	30.0%^a, b^	<0.001	52.6%^a, b^	39.8%^a^	0.003
Convenient	54.7%^a, b^	34.9%^a^		59.1%^a^	29.7%^b^		50.2%^a, b^	40.2%^a^	
Less convenient	56.8%^b^	34.8%^a^		59.7%^a^	30.9%^a, b^		53.6%^b^	39.2%^a^	
Inconvenient	49.1%^a^	44.2%^b^		55.0%^a^	37.8%^a, b^		43.0%^a^	50.9%^b^	
Highly inconvenient	50.7%^a, b^	43.3%^a, b^		48.0%^a^	43.0%^a^		53.5%^a, b^	43.6%^a, b^	
**Comorbidities**									
<1	36.6%^a^	2.0%^a^	<0.001	33.7%^a^	1.4%^a^	<0.001	40.5%^a^	2.8%^a^	<0.001
≥1	56.2%^b^	38.4%^b^		60.3%^b^	33.6%^b^		52.1%^b^	43.2%^b^	
**ADL disability**									
No	58.1%^a^	31.2%^a^	<0.001	60.7%^a^	27.0%^a^	<0.001	55.3%^a^	35.6%^a^	<0.001
Yes	13.7%^b^	86.2%^b^		17.1%^b^	82.6%^b^		11.1%^b^	88.9%^b^	
**Living southern or northern China**									
Northern China	47.9%^a^	45.0%^a^	<0.001	53.4%^a^	38.9%^a^	<0.001	43.3%^a^	50.2%^a^	<0.001
Southern China	56.9%^b^	32.3%^b^		59.2%^b^	28.3%^b^		54.3%^b^	36.8%^b^	

ADL, activities of daily living.

The superscripts a, b and c indicate the difference in the prevalence of pre-frailty and frailty among different groups (adjusted p-values).

Multinomial regression analysis revealed that female, increased age, illiteracy, living alone, hospitalization in the past 1 year, difficult financial situation, comorbid chronic diseases, ADL disability, and north of residence were risk factors for frailty and pre-frailty in older adults with asthma (Table 3).

**Table 3:** Factors associated with frailty and pre-frailty of older adults with asthma by multinomial logistic regression.

**Variables**		**Pre-frailty vs. robust**				**Frailty vs. robust**			
		**OR**	**95%CI**		***p*** **-value**	**OR**	**95%CI**		***p*** **-value**
			**Lower**	**Upper**			**Lower**	**Upper**	
Sex	Male	1 (ref)
	Female	1.1094	0.909	1.316	0.341	1.423	1.163	1.742	<0.001
Age (years)	60–69	1 (ref)
	70–79	1.364	1.126	1.652	0.001	1.618	1.313	1.993	<0.001
	≥80	1.830	1.388	2.412	<0.001	2.469	1.833	3.326	<0.001
Urban or rural area	Urban	1 (ref)
	Rural	1.250	1.052	1.482	0.011	1.211	1.004	1.460	0.046
Marriage	Married	1 (ref)
	Widowed	0.938	0.740	1.189	0.595	0.945	0.731	1.221	0.663
	Divorced	1.249	0.444	3.512	0.674	0.870	0.278	2.723	0.811
	Unmarried	1.632	0.752	3.543	0.595	1.056	0.452	2.456	0.901
Education	Non-illiterate	1 (ref)
	Illiterate	1.341	1.090	1.650	0.006	1.555	1.243	1.944	<0.001
Ethnicity	Han	1 (ref)
	Others	0.972	0.704	1.343	0.863	0.769	0.538	1.099	0.149
Living alone	No	1 (ref)
	Yes	4.810	3.298	7.014	<0.001	8.691	5.848	12.917	<0.001
Medical checkup within 1 year	Yes	1 (ref)
	No	0.927	0.782	1.100	0.385	1.021	0.370	2.820	0.968
Hospitalized within 1 year	No	1 (ref)
	Yes	1.609	1.342	1.929	<0.001	2.670	2.198	3.244	<0.001
Economic status	Very rich	1 (ref)
	Rich	1.608	0.772	3.351	0.205	1.371	0.586	3.209	0.466
	Adequate	2.175	1.063	4.448	0.033	2.257	0.986	5.165	0.054
	Poor	4.077	1.948	8.531	0.0002	6.658	2.849	15.558	<0.001
	Very poor	9.953	3.912	25.326	<0.001	33.890	12.074	95.126	<0.001
Medical reimbursement	Very convenient	1 (ref)
	Convenient	1.034	0.854	1.252	0.729	1.026	0.832	1.265	0.809
	Less convenient	1.203	0.936	1.547	0.149	1.122	0.854	1.474	0.408
	Inconvenient	1.328	0.828	2.130	0.239	1.569	0.951	2.588	0.078
	Very inconvenient	1.654	0.771	3.549	0.197	1.846	0.829	4.110	0.133
Co-morbidities	<1	1 (ref)
	≥1	25.971	21.077	32.001	<0.001	556.186	309.531	999.391	<0.001
ADL disabilities	No	1 (ref)
	Yes	49.491	6.632	369.356	<0.001	613.664	81.615	4,614.157	<0.001
Medicare	Yes	1 (ref)
	No	0.976	0.374	2.547	0.961	1.021	0.370	2.820	0.968
Living in southern or northern China	Southern	1 (ref)
	Northern	1.453	1.171	1.803	<0.001	2.229	1.772	2.804	<0.001

ADL, activities of daily living; CI, confidence interval; OR, odds ratio.

## Discussion

Asthma has long been recognized as a disease that is prevalent in adolescents. Our study showed a self-reported asthma prevalence of 4.5% in older adults, indicating that the prevalence of asthma in older adults is not low. Data from the 2010–2012 China Epidemiological Survey of Asthma Prevalence and Risk Factors study showed that the prevalence of asthma increased with age, with 2.26% of people aged 61–70 years and 3.10% of people aged ≥71 years (2), and data from the China Adult Lung Health Study from 2012 to 2015 showed that the prevalence of asthma was 6.0% in people aged 60–69 years and 7.4% in people aged ≥70 years (3). The above study showed that asthma prevalence was not low among older adults in China. Foreign epidemiological data showed that the prevalence of asthma in people aged 65 years or over was 4%−15% (1, 16). Asthma surveillance data released by the Centers for Disease Control and Prevention show that the prevalence of asthma among older adults ≥65 years of age in the United States was 8.1% in 2010 (1). The 2017 Global Burden of Disease Study shows that the prevalence of asthma has decreased since 1990, from 3.9% in 1990 to 3.6% in 2017, but it remains the second most prevalent chronic respiratory disease after chronic obstructive respiratory disease, and asthma is also the second leading cause of death from chronic respiratory disease (4).

The rate of severe asthma and mortality in older adults with asthma is higher than in other age groups. The risk of severe and frequent acute exacerbations, resulting in frequent hospitalizations or emergency room visits, significantly increases the direct medical costs of older adults with asthma (5). Prevention and early management of asthma, a common non-communicable disease in older adults, is also one of the key goals for achieving healthy aging, and studies of frailty as an assessment indicator of biological aging have shown a significant increase in negative clinical events in frail older adults (10); therefore, studying the frailty status and risk factors of older asthmatics can help inform public health policymakers to reduce cognitive and physical decline and prevent frailty and disability in older asthmatics.

Our large cross-sectional national study used a stratified, multi-stage, size-proportional probability sampling and a final-stage equal-probability sampling design with a sample covering 31 provinces, municipalities, and autonomous regions in mainland China with different geographic regions and economic development status, and the age distribution, gender, and urban-rural ratios of older adults in the obtained sample were consistent with the demographic characteristics of older adults in the 2015 China Population Survey, ensuring national representativeness. Our study accurately reports the prevalence of frailty and pre-frailty in older Chinese patients with asthma. Our study found a high age-sex standardized prevalence of frailty in older adults with asthma of 35.8% (95% CI 34.8%−36.7%) and an age-sex standardized prevalence of pre-frailty of 54.5% (95% CI 53.5%−55.5%), with a 3.3-fold increase in the prevalence of frailty in older adults with asthma compared to older adults without asthma. The results of our study are generally consistent with those of a small study who found a 37% prevalence of frailty among 203 older outpatients with asthma aged 60 years or older (17), and we report a higher prevalence of frailty among older adults with asthma than a Brazilian cohort study that reported 13% of older patients with current asthma had frailty in 2015 (18). Chronic inflammation in asthmatics is not only present in the respiratory tract but is systemic, characterized by increased levels of peripheral blood eosinophils, immunoglobulin E and type 2 cytokines. Recent studies have found that chronic systemic inflammation is associated with the development of frailty and that chronic systemic inflammation in older adults with asthma may be an important cause of frailty (19–21). Our study showed that the proportion of older asthmatics who never exercised was significantly higher than that of older adults without asthma, and that the reduction in exercise also resulted in older asthmatics being more likely to develop sarcopenia, one of the key factors in the development of frailty syndromes. Until the recent advent of biologic agents, oral corticosteroids (OCS) have been the key controller medication for the treatment of severe intractable asthma. Ryu et al. reported a higher prevalence of frailty in older adults with asthma with longer lifetime OCS exposure (33% of patients with no lifetime OCS use, 59% of low-dose users, and 68% of high-dose users; *p* < 0.005 for trend). Suggesting that lifetime cumulative OCS exposure was associated with a high prevalence of weakness and muscle weakness (17). Although OCS is effective for asthma, it causes side effects including osteoporosis, fractures, diabetes, obesity, cardiovascular disease, and infections that may promote the onset of frailty. These aforementioned factors may promote the development of frailty in older adults with asthma.

Our study found that being female, increasing age, illiteracy, living alone, hospitalization in the past 1 year, economic hardship, comorbid chronic diseases, ADL disability, and living in northern China were risk factors for frailty and pre-frailty in older asthmatic patients. Studies have shown that aging is an independent risk factor for the onset of frailty, as older adults age, several physiological systems throughout the body undergo degenerative changes, the reserve function of various organs decreases, and the risk of frailty increases (7, 22). Decreased estrogen levels in older women lead to a decrease in muscle strength and a negative impact on neuromuscular function and postural stability, leading to an increased incidence of frailty in older women (23). Educational attainment is highly correlated with income, and difficult economic status makes it difficult to obtain adequate medical care, which increases the risk of frailty (24, 25). Older asthmatics who are living alone are more likely to experience loneliness and depression due to decreased family support, leading to an increased risk of frailty (26). Older adults with asthma have multiple co-morbidities and these chronic diseases contribute to the increased risk of frailty in older adults with asthma (27). Therefore, in the management of older adults with asthma, attention should be paid to identifying risk factors for frailty and providing early targeted interventions to reduce or delay the risk of frailty in older adults with asthma.

Compared with male older adults with asthma, our study found that female older adults with asthma were older, had higher rates in the advanced age group, higher rates of illiteracy, widowhood, living alone, comorbid chronic diseases, disability, and hospitalization in the past 1 year, which are factors associated with higher prevalence of frailty and pre-frailty in female older adults with asthma than in men, and the findings of our study provide information for policy makers to take appropriate measures to reduce and prevent the occurrence of frailty in female asthmatics.

Our study also found that the prevalence of frailty was higher among older adults with asthma living in the north than in the south. We found higher rates of female, widowed, no physical examination in the past 1 year, difficult financial status, inconvenient medical reimbursement, comorbid chronic diseases and ADL disability among older adults with asthma in northern China than in southern China. Differences in the demographic characteristics of older adults with asthma and risk factors for frailty in northern and southern China may explain the higher prevalence of frailty among older adults with asthma in the north than in the south. These findings provide information for policy makers to take appropriate measures to reduce and prevent the occurrence of frailty in asthmatic patients in northern China.

Limitations: firstly, the self-reported diagnostic information of asthma in this study may be subject to recall bias, secondly, there is a lack of information on the course and extent of disease in older adults with asthma, and at least two phenotypes exist in older adults with asthma; long-term asthmatics have more severe airflow limitation and are less fully reversible than asthmatics with late onset asthma. Third, smoking is an important risk factor for chronic airway disease, and this study did not assess the association between smoking and frailty in older adults with asthma. Fourth, this study was a cross-sectional study and could not determine the causal relationship between associated factors and frailty in older adults with asthma.

In conclusion, the prevalence of frailty and pre-frailty in older adults with asthma is very high, and frailty assessment should become a routine in the management of older adults with asthma, and attention should be paid to the early identification of risk factors for frailty in older adults with asthma and targeted interventions to prevent and delay the onset of frailty in older adults with asthma.

## Data availability statement

The original contributions presented in the study are included in the article/Supplementary material, further inquiries can be directed to the corresponding author.

## Ethics statement

The studies involving human participants were reviewed and approved by the National Bureau of Statistics (No. [2014] 87) and the Ethics Committee of Beijing Hospital (2021BJYYEC-294-01). The patients/participants provided their written informed consent to participate in this study.

## Author contributions

X-zZ and NJ wrote the various drafts of the manuscript. X-zZ, L-bM, and JS conducted the statistical analyses. CZ, Y-yL, J-bH, HL, XQ, HW, XH, D-sW, and J-yL participated in data interpretation. D-pL, Q-xZ, JL, and X-zZ conceived and designed this study. X-zZ, NJ, L-bM, CZ, Y-yL, J-bH, JS, HL, XQ, HW, XH, D-sW, J-yL, Q-xZ, JL, and D-pL were revised the drafts of the manuscript for important scientific content. D-pL was the guarantor of this work and, as such, had full access to all the data in the study and takes responsibility for the integrity of the data and the accuracy of the data analysis. All authors gave final approval of the version to be published.
