# Supplementary material for: Epidemiological status and associated factors of frailty and pre-frailty in older adults with asthma in China: A national cross-sectional study
Source: Front Public Health. 2023 Mar 3;11:1136135. doi: 10.3389/fpubh.2023.1136135 (PMC10022817; doi:10.3389/fpubh.2023.1136135)
Supplement: Supplementary file 1 [file Table_1.DOCX]

Supplemental Table 1. Demographics of the Chinese older adults with asthma in 2015, and risk factors for frailty, by North-South subgroups.

|  | Total  (n=9416) | Northern  (n=2382) | Southern  (n=7034) | P for difference |
| --- | --- | --- | --- | --- |
| Proportional of participants | 100% | 25.3% | 74.7% |  |
| Sex (Women ) | 49.3% | 54.1% | 47.7% | <0.001 |
| Age (years) | 69·7±7·8 | 71.0±8.0 | 72.1±8.1 | <0.001 |
| Age group |  |  |  | <0.001 |
| 60-69 | 44.4% | 49.4% a | 42.4% b |  |
| 70-79 | 35.8% | 33.0% a | 36.8% b |  |
| ≥80 | 19.8% | 17.5% a | 20.5% b |  |
| Urban or rural area (Urban) | 41.4% | 42.2% | 41.1% | 0.369 |
| Education (Illiteracy) | 36.9% | 31.8% | 38.6% | <0.001 |
| Marital status |  |  |  | <0.001 |
| Married | 65.9% | 64.6% a | 66.3% a |  |
| Widowed | 31.5% | 33.7% a | 30.7% b |  |
| Divorced | 0.8% | 0.8% a | 0.8% a |  |
| Unmarried | 1.9% | 0.8% a | 2.2% b |  |
| Ethnicity (Non-Han) | 7.0% | 7.9% | 6.7% | 0.059 |
| Living alone | 16.2% | 15.0% | 16.5% | 0.085 |
| Health checkup within 1 year | 57.8% | 51.8% | 59.8% | <0.001 |
| Hospitalized within 1 year | 44.6% | 43.3% | 45.0% | 0.163 |
| Economic status |  |  |  | <0.001 |
| Very rich | 0.8% | 0.8% a | 0.8% a |  |
| Rich | 12.1% | 10.5% a | 12.6% b |  |
| Adequate | 55.1% | 51.9% a | 56.2% b |  |
| Poor | 26.6% | 29.1% a | 25.7% b |  |
| Very poor | 5.5% | 7.8% a | 4.7% b |  |
| Medicare (No) | 1.0% | 1.0% | 1.0% | 0.724 |
| Convenience of medical cost reimbursement |  |  |  | <0.001 |
| Highly convenient | 30.6% | 27.6% a | 31.7% b |  |
| Convenient | 43.4% | 44.5% a | 43.0% a |  |
| Less convenient | 18.9% | 18.5% a | 19.1% a |  |
| Inconvenient | 4.9% | 6.3% a | 4.5% b |  |
| Highly inconvenient | 2.1% | 3.1% a | 1.8% b |  |
| Comorbidities (≥1) | 92.0% | 93.5% | 91.6% | 0.003 |
| ADL disability | 7.8% | 12.1% | 6.4% | <0.001 |

Abbreviations: ADL, activities of daily living

Notes:The letters a, b indicate the difference in the demographics among different subgroups (adjusted p-values)
